# Management of femoral large-bore access and closure during microaxial flow pump-supported interventions

**DOI:** 10.3389/fcvm.2026.1816473

**Published:** 2026-05-15

**Authors:** Rocco Vergallo, Marco Lombardi, Andrea Macchione, Fabio Pescetelli, Stefano Benenati, Matteo Vercellino, Gioel G. Secco, Nieves Gonzalo, Javier Escaned, Italo Porto

**Affiliations:** 1 Department of Internal Medicine and Medical Specialties (DIMI), Università di Genova, Genova, Italy; 2 Cardiothoracic and Vascular Department (DICATOV), IRCCS Ospedale Policlinico San Martino, Genova, Italy; 3 Interventional Cardiology Unit, AOU SS Antonio e Biagio e Cesare Arrigo, Alessandria, Italy; 4 Department of Translational Medicine, University of Eastern Piedmont, Novara, Italy; 5 Interventional Cardiology Unit, Hospital Clínico San Carlos IDISSC and CIBER-CV, Complutense University of Madrid, Madrid, Spain

**Keywords:** hemodynamic support, Impella, large-bore access, microaxial flow pump, post-closure, pre-closure, vascular closure device, vascular complications

## Abstract

Microaxial flow pump (mAFP) is increasingly employed for hemodynamic support in both cardiogenic shock (CS) and high-risk percutaneous coronary interventions (HR-PCI). However, its implantation requires large-bore arterial access, which carries a substantial risk of vascular complications, translating into higher morbidity, mortality, and healthcare costs. Optimizing outcomes depends on safe vascular access and effective closure strategies. Ultrasound- and angiography-guided puncture, often complemented by advanced imaging, enhance the safety of femoral access. In the HR-PCI setting, pre-closure techniques are generally feasible and advisable as a prophylactic strategy. Conversely, in CS patients, where pre-closure is frequently not achievable, post-closure techniques during mAFP removal play a pivotal role. This review provides a contemporary, practical framework to manage femoral large-bore access and closure in patients undergoing mechanical circulatory support with mAFP.

## Introduction

Percutaneous mechanical circulatory support (MCS) devices are increasingly employed to provide hemodynamic support both in the acute setting of cardiogenic shock (CS) and electively during protected high-risk percutaneous coronary interventions (HR-PCI) (1–4).

Among MCS, the microaxial flow pump (mAFP) has been gaining a broad clinical uptake, supported by an expanding body of evidence across a spectrum of indications (5, 6). The Impella device (Abiomed, Inc., part of Johnson & Johnson MedTech) is a continuous-flow mAFP positioned across the aortic valve that unloads the left ventricle (LV) and provides systemic hemodynamic support with flows of up to 5.5 L/min. While the transfemoral Impella CP is typically implanted percutaneously in the catheterization laboratory, axillary or femoral placement of the larger Impella 5.0/5.5 generally requires a surgical cut-down (7, 8). The rate of vascular complications varies across the registries (9–11), largely reflecting differences in patient profiles and clinical context (12). Managing these complications remains challenging, as they contribute not only to increased cardiovascular morbidity and in-hospital mortality, but also to prolonged hospital stays and higher healthcare costs (13).

The aim of this review is to provide a contemporary and pragmatic overview of vascular access management during mAFP support for elective or emergent cases, from optimal arterial puncture to safe device removal, with a focus on strategies to prevent and mitigate vascular complications. This review has a primary focus on left-sided mAFP and, specifically, on the Impella CP device.

## Incidence and impact of access-site complications during mAFP support

Access-site complications represent a major clinical concern in patients undergoing mAFP support. In a cohort study from the US National Inpatient Sample (NIS) database including 221,700 patients receiving MCS, the incidence of arterial access complications was highest with extracorporeal membrane oxygenation (ECMO, 15.8%), followed by mAFP (5.6%) and intra-aortic balloon pump (IABP, 3.0%). The study population encompassed both elective procedures (HR-PCI, 17%) and acute presentations [acute myocardial infarction (AMI), acute heart failure, cardiogenic shock] (13). Among patients with vascular complications, in-hospital mortality was highest in those supported with ECMO (56%), followed by mAFP (34%) and IABP (26%). Notably, in all subgroups access-site complications were associated with excess mortality. In mAFP cases, specifically, in-hospital mortality was 10% higher among patients with vascular complications compared to those without (34% vs. 24%, *p* < 0.001) (13). Importantly, vascular complications also drive up resource utilization and healthcare costs (∼$103k vs. ∼$63k, *p* < 0.001), regardless of the MCS device used. Interestingly, IABP patients with vascular complications generated higher costs than mAFP patients without vascular complications (∼$85 vs. ∼$62k) (13). These data highlight the clinical and economic relevance of access-site complications, and underscore the importance of prevention during both implantation and removal. The risk of complications may further increase with prolonged mAFP support. A PROTECT III substudy showed a higher incidence of hematoma (12% vs. 7%, *p* = 0.01) and limb ischemia (6% vs. 1%, *p* < 0.0001) in patients undergoing prolonged mAFP support than in those in whom mAFP was used intraprocedurally and removed after HR-PCI (14). Of note, patient factors like peripheral arterial disease (PAD), female sex, anemia and shock presentation have been identified as independent risk factors for mAFP access complications (15).

Finally, registry data emphasize the influence of clinical context and timing of mAFP insertion on the occurrence of vascular complications. In the IMP-IT registry, which enrolled 406 patients (56% CS, 44% HR-PCI), limb ischemia was more frequent in CS patients (13% vs. 3%, *p* < 0.0001), while the rate access-site bleeding (∼10% of cases) was not different between the two groups (12). As compared with a bailout mAFP insertion, a pre-procedural device insertion was associated with a higher one-year survival both in patients with CS related to AMI and in those undergoing HR-PCI. Importantly, pre-procedural implantation (as opposed to bailout insertion) was associated with higher 1-year survival in both CS and HR-PCI patients, and with reduced rates of mortality, heart failure re-hospitalization, and need for advanced therapies among HR-PCI patients (14). Conversely, bailout insertion was linked to increased rates of life-threatening or severe bleeding in both CS (16% vs. 7%, *p* = 0.1) and HR-PCI patients (9% vs. 1%, *p* = 0.02) (16).

## Pre-closure strategies in elective mAFP cases

Prophylactic (planned) mAFP support in hemodynamically stable patients undergoing HR-PCI may help maximize the long-term clinical benefit of complex procedures in selected patient (9, 17). However, evidence is largely derived from observational studies and registries (9, 10, 12, 18), as the PROTECT II trial did not demonstrate a significant advantage of mAFP over IABP on clinical outcomes (19). More recently, a meta-analysis reported a significant improvement in LV systolic function at 6-month follow-up among HR-PCI patients treated with mAFP (20). Additional evidence is anticipated from the ongoing PROTECT IV trial (NCT04763200) (21).

**Figure 1:**
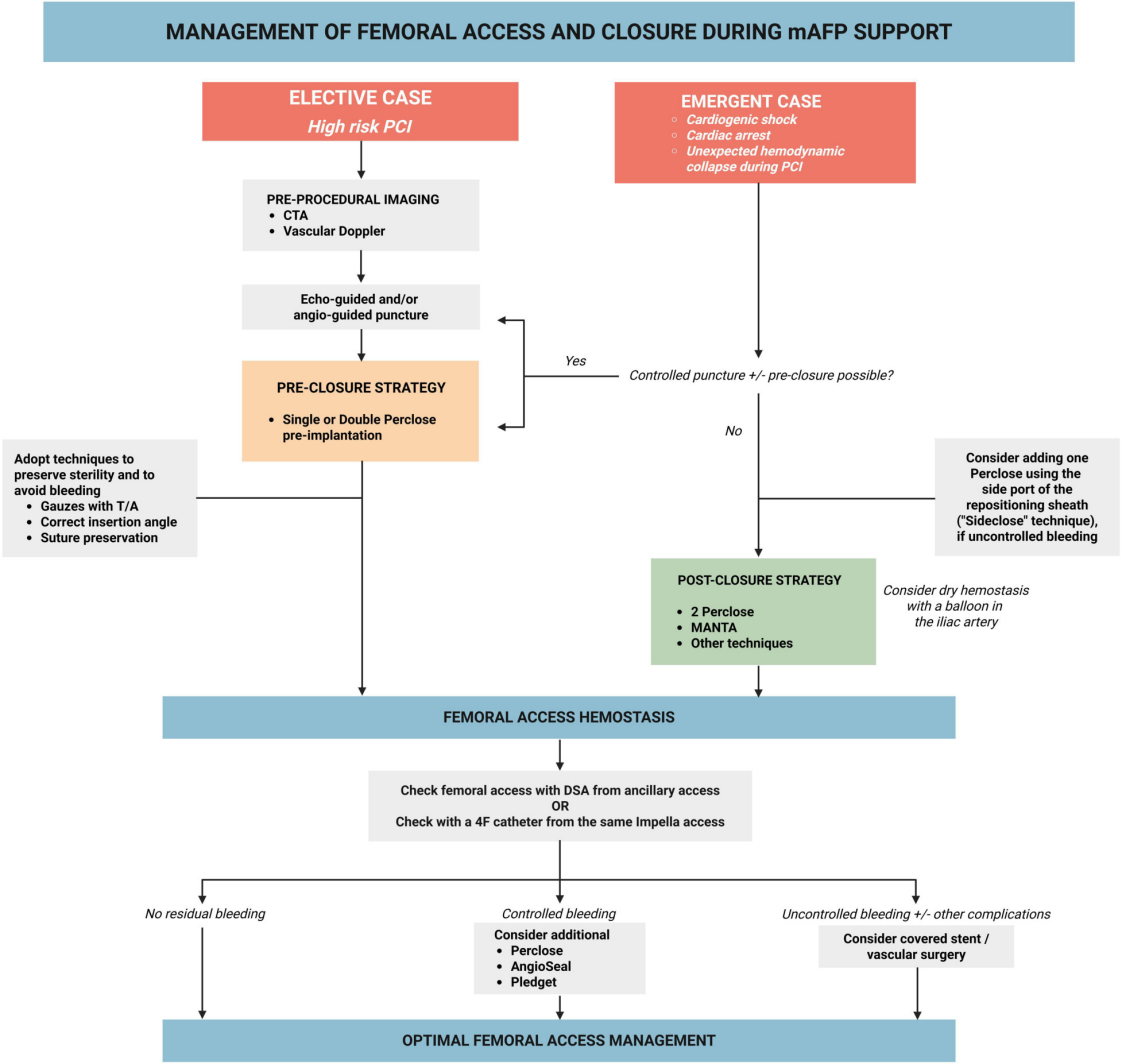
Proposed algorithm for the management of femoral large-bore access and closure in patients undergoing microaxial flow pump (mAFP)-supported interventions. Proposed algorithm for the management of femoral large-bore access and closure in patients undergoing mAFP-supported interventions in elective or emergent settings. mAFP, microaxial flow pump; PCI, percutaneous coronary intervention; CT, computed tomography; T/A, tranexamic acid.

In the prophylactic HR-PCI setting, pre-procedural assessment of the iliofemoral anatomy with CT angiography is advisable, as it facilitates selection of the most suitable vascular access site (Figure 1). Achieving safe femoral access often requires multimodality guidance, including fluoroscopy, angiography, and ultrasound (22–24). Ultrasound is particularly valuable to ensure central puncture of the common femoral artery at a healthy segment of the anterior wall, thereby avoiding either high (above the inferior epigastric artery) or low punctures (below the bifurcation of the superficial and profunda femoral arteries). Accurate vessel puncture represents the cornerstone for successful use of vascular closure devices (25).

Conversely, patients presenting with CS often require emergent large-bore access without prior anatomical information, in the absence of pre-procedural imaging. In such cases, the choice between a planned vs. bailout strategy is dictated by the perceived risk of hemodynamic collapse during PCI (26). O'Neill et al. compared outcomes between bailout and prophylactic mAFP support in a cohort of 1,028 HR-PCI patients (57 vs. 971, respectively). Bailout patients were more often women (51% vs. 27%, *p* = 0.0002), had a higher baseline LVEF (40% vs. 30%, *p* < 0.0001), and a lower prevalence of heart failure (42% vs. 57%, *p* = 0.0385) and left main disease (40% vs. 56%, *p* = 0.0250). Despite this, both unadjusted and adjusted in-hospital mortality were markedly higher in the bailout group (49% vs. 4% and 58% vs. 4%, respectively; *p* < 0.0001) (27). For this reason, whenever possible, controlled femoral artery puncture and pre-closure should be sought also in the emergent setting.

In the elective HR-PCI setting, pre-closure of the large-bore femoral access should be standard practice, as it facilitates reliable haemostasis at the time of device removal and reduces the risk of vascular complications (Figure 1). Pre-closure can be achieved using one/two Perclose devices (ProGlide or ProStyle, Abbott Vascular, USA) (28).

### Technical aspects of pre-closure

Pre-closure involves deploying a vascular closure device after puncture (before upsizing to the large 14F Impella sheath) so that sutures are in place for later removal. In practice, the dual Perclose technique has gained popularity—typically one suture device is placed at ∼10 o'clock and a second at ∼2 o'clock prior to inserting the 14 Fr Impella sheath. Alternatively, some operators prefer placing two Perclose sutures in parallel without device rotation, in order to minimize the risk of suture entanglement (24, 29). During this maneuver, special care should be taken to avoid pulling on the locking (white) suture, whereas gentle traction on the rail (non-locking) suture may be applied to facilitate hemostasis and allow smooth advancement of the 14F sheath (30).

### mAFP removal and hemostasis

**Figure 2:**
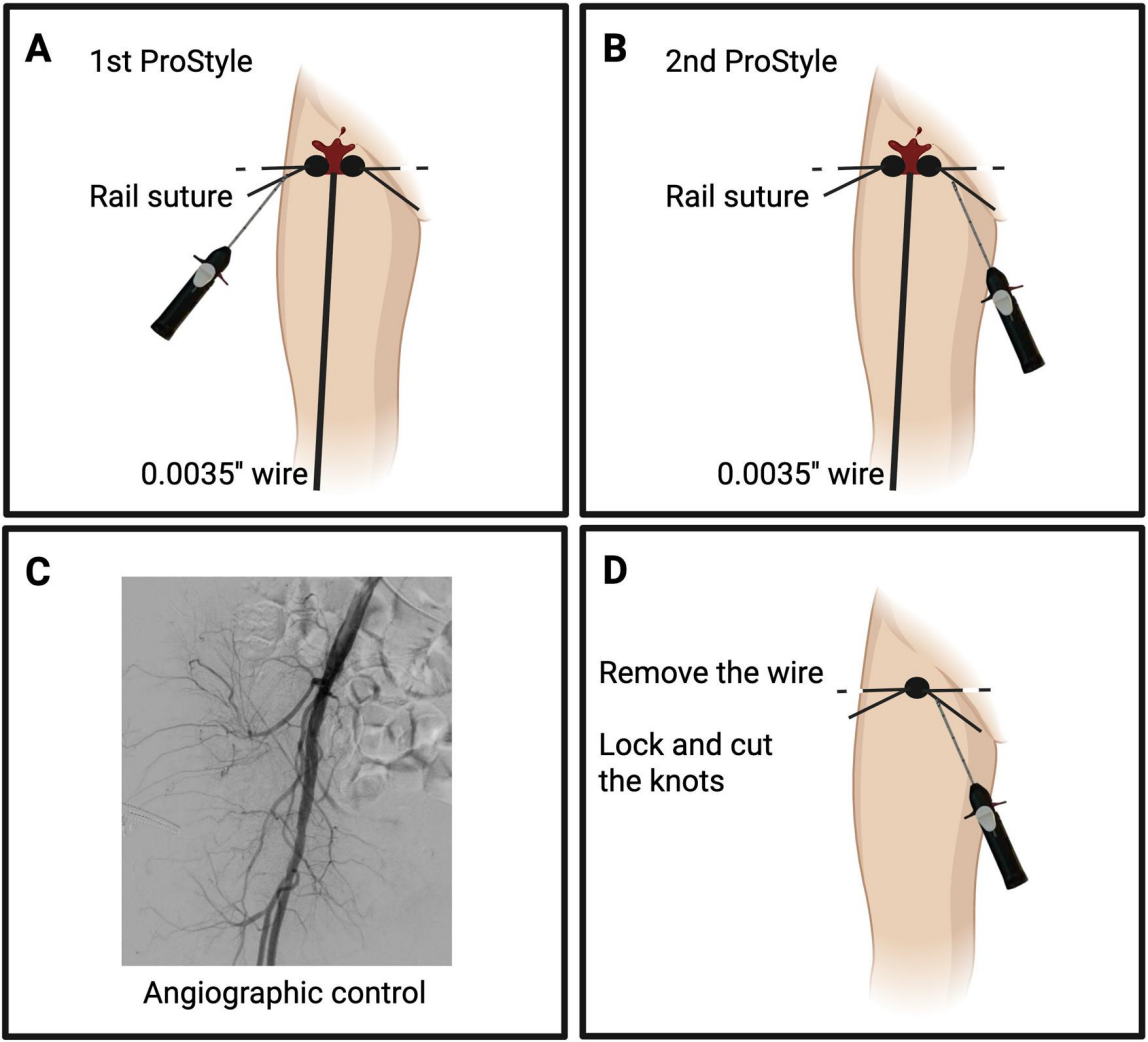
Stepwise femoral closure following mAFP removal using the pre-closure technique with two Perclose (ProStyle, Abbott Vascular, USA). Panel **(A)**, deployment and initial securing of the first Perclose using the snare knot pusher; Panel **(B)**, deployment and tightening of the second Perclose device; Panel **(C)**, selective angiographic control may be considered to confirm hemostasis and exclude vascular complications; Panel **(D)**, the 0.035″ guidewire is removed, Perclose knots are locked in sequence, and the suture tails are cut.

Once the procedure is completed and mAFP support is no longer required, the preplaced sutures are deployed to achieve closure. Typically, the first Perclose suture is secured using the knot pusher, followed by tightening of the second (30). After arteriotomy closure, the access site should be evaluated angiographically, ideally with digital subtraction angiography (DSA), given its higher sensitivity for detecting subtle bleeding, using either contralateral femoral or radial access (Figure 2). In the setting of single-access mAFP-protected PCI, integrity of the arteriotomy can alternatively be verified by inserting a 4F diagnostic catheter (e.g., JR4) over a 0.035″ guidewire (31). This simple technique allows confirmation of hemostasis without needing a separate access. If pre-closure fails, a regular (or, better, a stiff core) 0.035″ wire can be advanced to enable an additional Perclose. Once satisfactory hemostasis is achieved, the sutures are locked in sequence and cut. Alternatively, an AngioSeal (Terumo, Japan) can be deployed (25), as part of a hybrid suture–plug strategy supported by observational and randomized studies (32–34). Prior to Angio-Seal deployment, a second safety wire might be maintained through a temporary 5–8 Fr sheath to preserve access and allow alternative closure strategies in case of device failure. Once the AngioSeal is deployed, the guidewire is lost; therefore, it is essential to verify the suitability of the AngioSeal prior to deployment. To assess this, an 8F sheath can be advanced—if hemostasis is achieved with the sheath in place, it indicates that an 8F AngioSeal will be sufficient.

**Figure 3:**
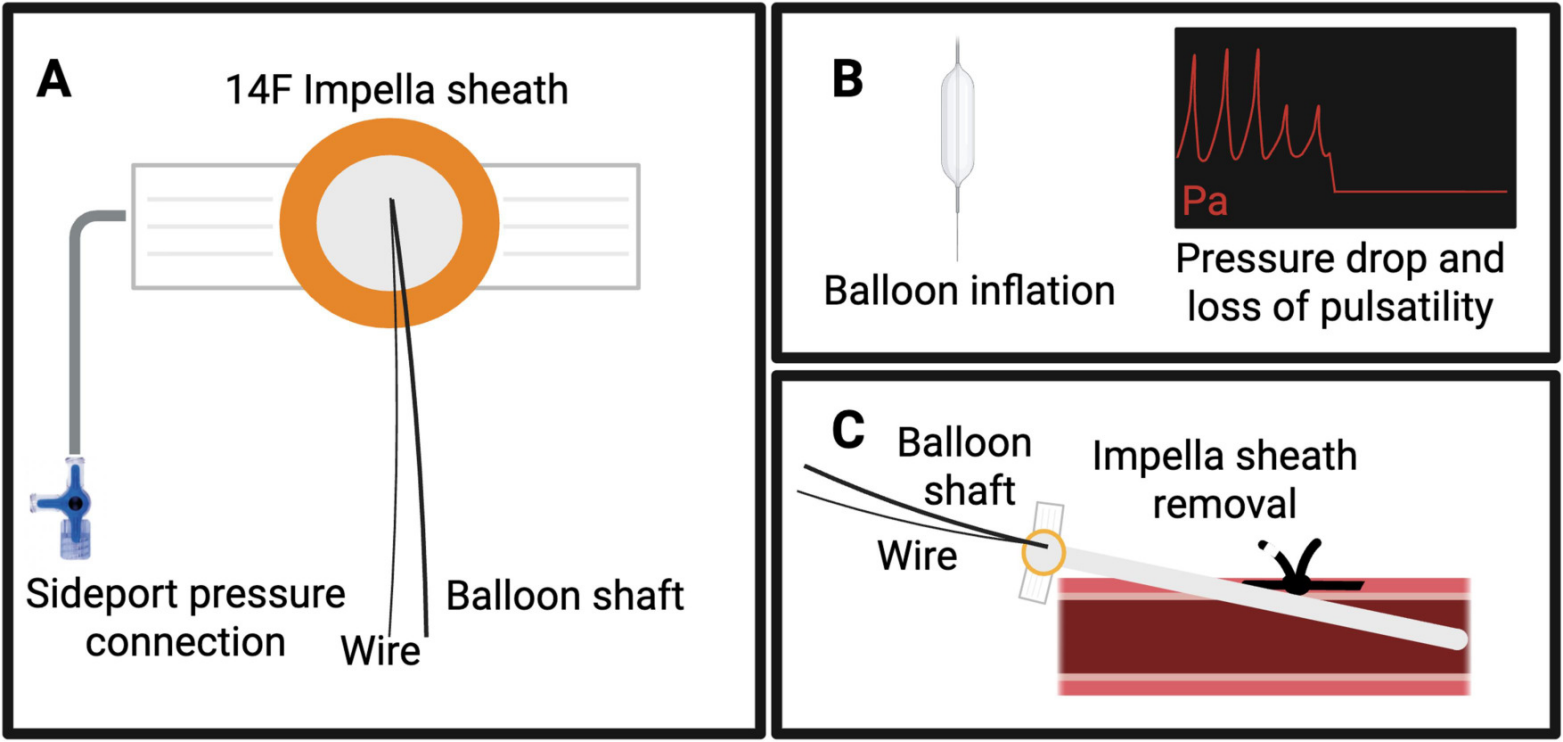
Single-Access Dry-closure technique during Impella mAFP-supported interventions. Panel **(A)**, advance the peripheral balloon (sized at 1:1 ratio to the external iliac artery) over a 0.035″ or a 0.018″ stiff wire through the 14F Impella sheath and connect the sheath sideport to a pressure transducer; Panel **(B)**, inflate the balloon until the pressure waveform from the sheath sideport is no longer visible; Panel **(C)**, withdraw the 14F Impella sheath over the balloon shaft and secure the Perclose (ProStyle, Abbott Vascular, USA) sutures for effective closure.

Several adjunctive techniques, involving an ancillary access site, have been described to maintain hemostatic control while preserving the option for endovascular bailout procedures. Depending on patient anatomy and operator preference, balloon-assisted (“dry”) hemostasis can be performed through a contralateral femoral or radial access, or even via the ipsilateral artery. The occlusion balloon should be positioned in the external iliac artery (rather than at the common femoral artery) to avoid interference, and intermittent deflation may be required to facilitate device advancement and optimal positioning. Importantly, in procedures performed with a single arterial access [Single-access for High-risk PCI (SHiP) technique] (35), balloon-assisted closure may also be achieved through the same 14F mAFP sheath. This so-called Single-Access Dry-Closure Technique enables reliable closure while retaining the flexibility to escalate to further endovascular solutions (e.g., additional Perclose or AngioSeal deployment) in case of persistent bleeding (36). A step-by-step overview of the technique is shown in Figure 3. These techniques of “dry hemostasis” are even more useful in cases of post-closure, when controlling access bleeding might be more challenging.

## Prolonged mAFP support: retrieval of the 14F peel-away introducer and advancement of the repositioning sheath

**Figure 4:**
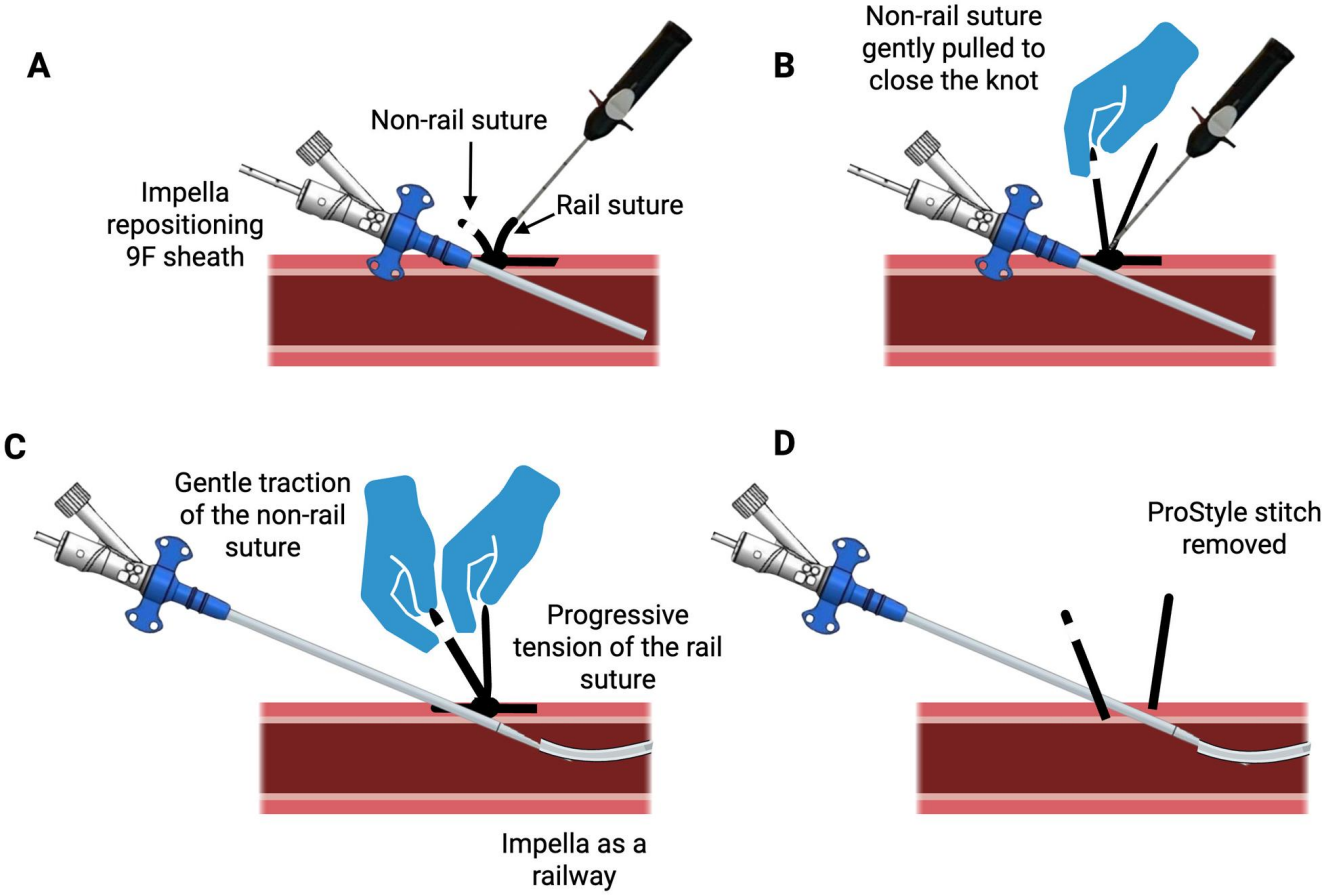
Impella mAFP repositioning sheath (9F) secured with the Preclose C-stitch technique. Panel **(A)**, Repositioning of Impella mAFP 9F sheath, next with the Perclose (ProStyle, Abbott Vascular, USA) knot pusher on the rail suture, the slipknot is advanced beneath the skin. Panel **(B)**, the non-rail suture (white suture) is gently pulled to close the knot around the 9F sheath. It is essential not to cut the suture tails to facilitate the breaking of the cinching stitch during Impella mAFP removal; Panel **(C)**, applying a gentle traction on the non-locking stitch (non-rail suture, white suture) while slowly and progressively increasing tension in the same direction as the locking stitch (rail suture); Panel **(D)**, ProStyle cinching stitch is carefully removed.

In patients requiring prolonged hemodynamic support after PCI, meticulous management of the access site is essential to preserve arterial integrity and minimize bleeding risk. A relevant technical aspect is the discrepancy between the 14F peel-away introducer sheath and the smaller 9–13F tapered “repositioning sheath”, which may predispose to peri-sheath oozing. Several strategies have been described to address this issue. One option is the pre-close cinching “C-Stitch” technique (37), in which two operators coordinate removal of the peel-away sheath while advancing the tapered repositioning sheath at a 45° angle over the mAFP catheter, used as a rail. Once positioned, the Perclose knot is secured around the repositioning sheath, and the mAFP catheter can be safely withdrawn under controlled tension on the Perclose suture. A step-by-step illustration is provided in Figure 4.

Adjunctive measures such as manual compression and local application of gauze soaked in tranexamic acid or adrenaline may further reduce oozing and bleeding (38). Another useful maneuver is the “pull-up stitching technique”, which involves advancing the repositioning sheath deep into the subcutaneous tissue, securing the Perclose knot snugly around it, and placing a gauze pad beneath the sheath to maintain the insertion angle. This approach helps stabilize the cannula and mitigate sheath-related oozing. When it is necessary to leave the mAFP in place, several techniques can help manage the Perclose sutures to maintain appropriate tension and prevent accidental closure. A common approach involves placing the sutures inside a short segment of tubing—typically cut from the removed mAFP introducer or from a femoral sheath used during access. The tubing helps organize and protect the sutures, which can then be secured using a mosquito clamp to avoid unintentional tightening or loss of suture control.

## Bail-out post-closure strategies in emergent mAFP cases

In emergency scenarios (e.g., cardiogenic shock, cardiac arrest, or hemodynamic collapse during elective PCI) or when prolonged mAFP support is anticipated, pre-closure may not be feasible (Figure 1). In these settings, alternative post-closure techniques become crucial to ensure safe device removal and minimize bleeding complications. Whenever possible, post-closure should be performed in the catheterization laboratory rather than at bedside, as this setting enables fluoroscopic guidance and the use of “dry hemostasis” techniques if required. Schott et al. reported lower bleeding rates with percutaneous mAFP removal in the cath lab compared with bedside manual or surgical removal (13% vs. 20% vs. 43%) (15).

A stepwise approach is recommended. Balloon-assisted closure is typically the first-line strategy, achieved by advancing a peripheral balloon from a contralateral femoral or transradial access and inflating it in the ipsilateral external iliac artery. This maneuver facilitates haemostasis during deployment of a vascular closure device and can also be used as a crossover balloon occlusion technique if residual bleeding persists.

The choice of ancillary access is relevant. Transradial access may allow effective endovascular management using long-shaft, 6 Fr-compatible balloons and selected stents; however, device compatibility is limited, and larger-profile devices typically require contralateral femoral access (24).

Importantly, ancillary access also allows for final angiographic control to confirm vessel patency, exclude extravasation, and detect potential complications such as stenosis, dissection, or perforation (26, 39). When dealing with a residual stenosis, balloon angioplasty and/or peripheral stent placement may be considered, and operators need to be aware that only a few stents are 6/7 Fr compatible (e.g., Zilver PTX, Cook Medical, Indiana or Eluvia stent, Boston Scientific, USA).

In case of persistent bleeding, endovascular bailout should be considered.

Covered stent implantation represents an effective option for sealing access-site bleeding. Balloon-expandable covered stents (e.g., VBX Stent Graft, W.L. Gore & Associates, AZ) provide greater radial strength and are generally preferred for precise deployment (i.e., common femoral artery), whereas self-expanding covered stents (e.g., Viabahn®, W.L. Gore & Associates, AZ) offer great flexibility and may be advantageous in more proximal or tortuous segments (i.e., external iliac artery) (40). However, their use is limited by device profile and sheath compatibility, as most currently available covered stents require ≥8–9 Fr systems and are not compatible with 6–7 Fr access. Accordingly, these devices are typically delivered through an appropriately sized sheath, and sheathless delivery is generally not feasible due to limited trackability and support. As a result, contralateral femoral access is often necessary to allow delivery of these devices.

Surgical repair remains the final option when percutaneous strategies fail (26).

### Post-closure with Perclose suture(s)

**Figure 5:**
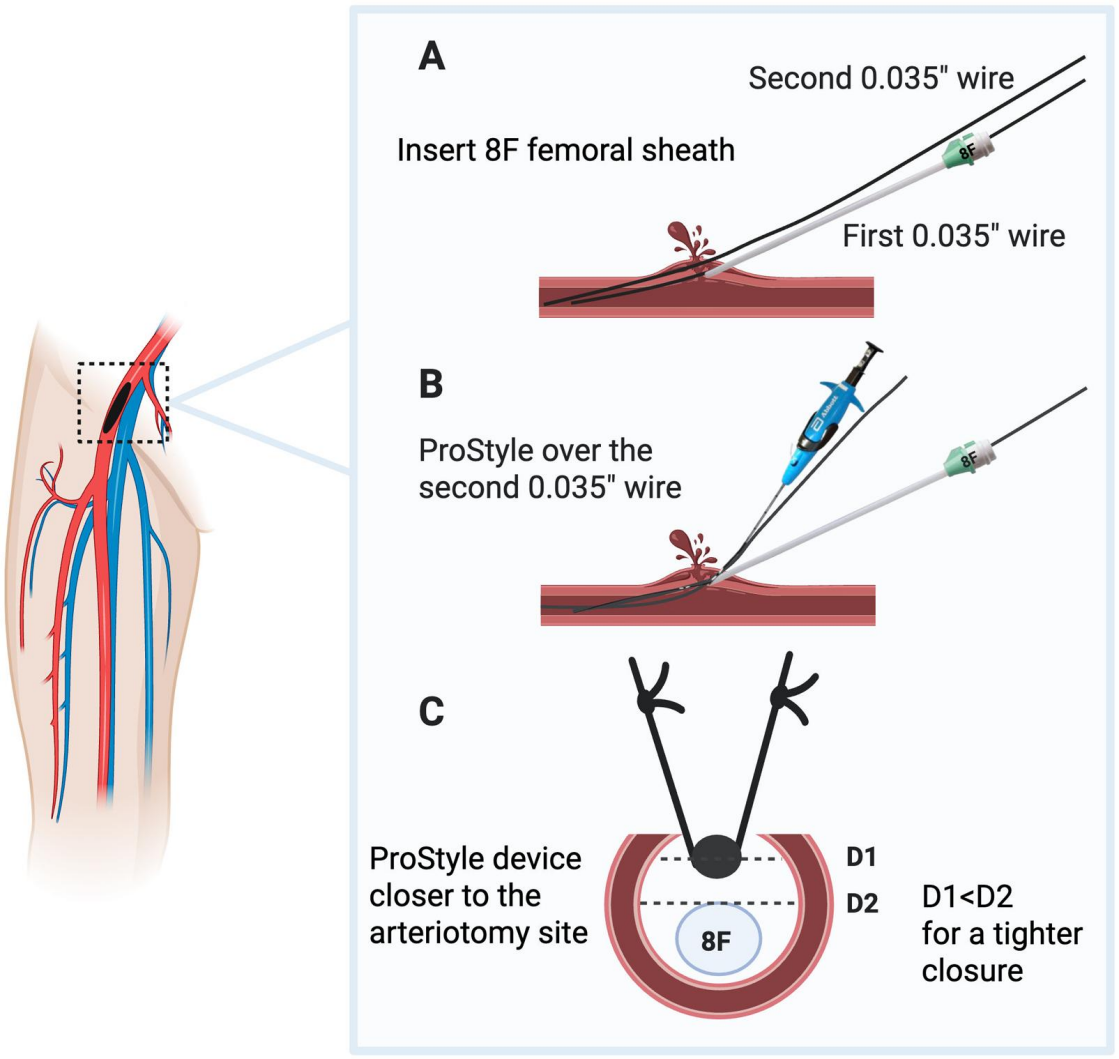
Post-closure technique with two Perclose (ProStyle, Abbott Vascular, USA) facilitated by an additional sheath. Post-closure of Impella mAFP access using a double Perclose technique can be facilitated by the use of a second 8–10F sheath. Panel **(A)**: an 8–10F sheath is placed over 0.035 wire introduced through the repositioning sheath. This additional step allows introducing a second 0.035″ wire through the 8–10F sheath, enabling a double-wire approach for the deployment of two Perclose. Panels **(B,C)**: the first Perclose is deployed while an 8-10F sheath is in place, so that the suture is delivered at a location where the arteriotomy is smaller, allowing for a more effective closure. Once the first Perclose is delivered, the suture can be tightened on the sheath, so that the second Perclose can be deployed to close the remaining 8–10F bore. D1, diameter 1; D2, diameter 2.

**Figure 6:**
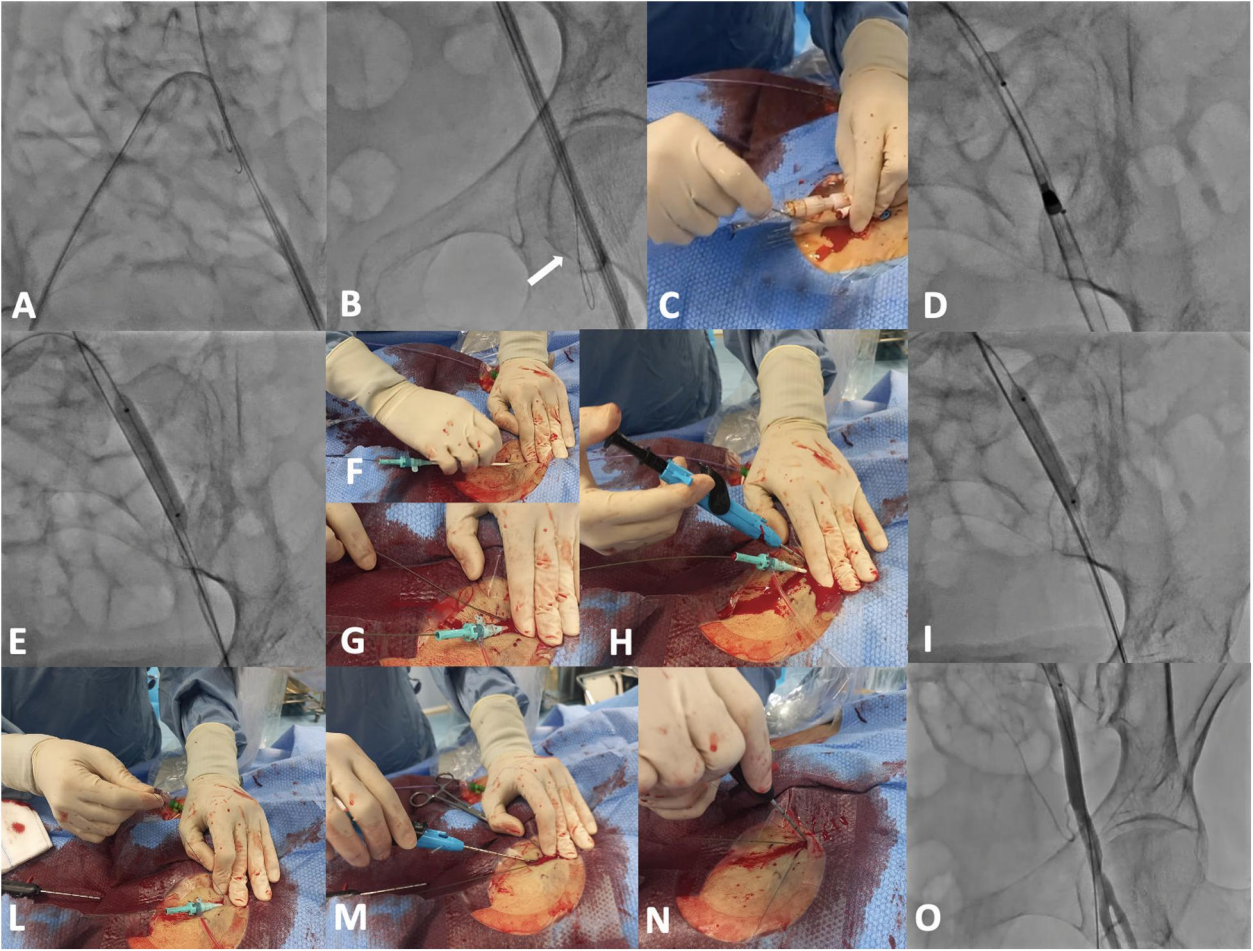
Representative case of post-closure technique using two Perclose (ProStyle, Abbott Vascular, USA) suture-based devices, facilitated by a second 8F sheath and balloon inflation in the external iliac artery (“dry-hemostasis”). Panel **(A)**, Cross-over with an internal mammary catheter 6F from contralateral femoral artery to the homolateral common iliac artery (Supplementary Video S1); Panel **(B)**, advancement of a 0.018″ wire through the external iliac artery distally to the superficial femoral artery (Supplementary Video S2); Panel **(C)**, after removing the sidearm stylet, a 0.035″ wire is inserted through the side port of the Impella sheath (Supplementary Video S3); Panel **(D)**, a balloon is positioned in the external iliac artery (sized 1:1) and the Impella catheter is pulled back in the common femoral artery; Panel **(E)**, the balloon is inflated (Supplementary Video S4) and Impella catheter is completely removed over the 0.035″ wire (Supplementary Video S5-S6); Panel **(F)**, an 8F sheath is inserted over the 0.035″ wire; Panel **(G)**, a second 0.035″ wire is inserted, and then externalized by pulling out the 8F sheath and reintroducing it over the other wire; Panel **(H)**, first ProStyle deployment; Panel **(I)**, balloon inflation facilitates hemostasis during these steps (“dry-hemostasis”) (Supplementary Video S7); Panel **(L)**, the first suture is tightened over the 8F sheath; Panel **(M)**, the 8F sheath is then removed and a second ProStyle is deployed to close the residual 8F bore; Panel **(N)**, the second suture is tightened, closing the arteriotomy; Panel **(O)**, angiographic control through the tip of the balloon (Supplementary Video S8). If necessary, an injection through a catheter advanced over the 0.018″ wire can be performed for a final angiographic control, before wire removal.

Suture-based closure after large-bore arteriotomy presents specific challenges, particularly the limited capacity to secure sufficient anterior wall tissue after insertion of a large sheath. To address this issue, a stepwise approach is recommended. A 0.035″ guidewire can be introduced via the side port of the Impella “repositioning sheath”, maintaining access while catheter is removed in an over-the-wire, rapid-exchange fashion. One or two Perclose devices can then be deployed depending on vessel size, anatomy, and operator preference. Use of an intermediate 8–10F sheath over the 0.035″ wire can facilitate this process (39). This sheath reduces the effective arteriotomy size, enables a double-wire technique for sequential Perclose deployment, and allows the first suture to be locked onto the sheath itself before completing closure of the residual bore (Figure 5). Post-closure with a single Perclose has been reported, with the option of a second Perclose as backup (41). Alternatively, after the first Perclose, a plug-based closure device (e.g., AngioSeal) may be deployed to complete hemostasis depending on operator preference and residual bleeding (42, 43). Figure 6 and Supplementary Video S1–S8 show the step-by-step procedure of Impella removal and the post-closure technique with two Perclose sutures and “dry hemostasis”.

### The “Sideclose” technique: a post-closure method for hemostasis during ongoing mAFP support

**Figure 7:**
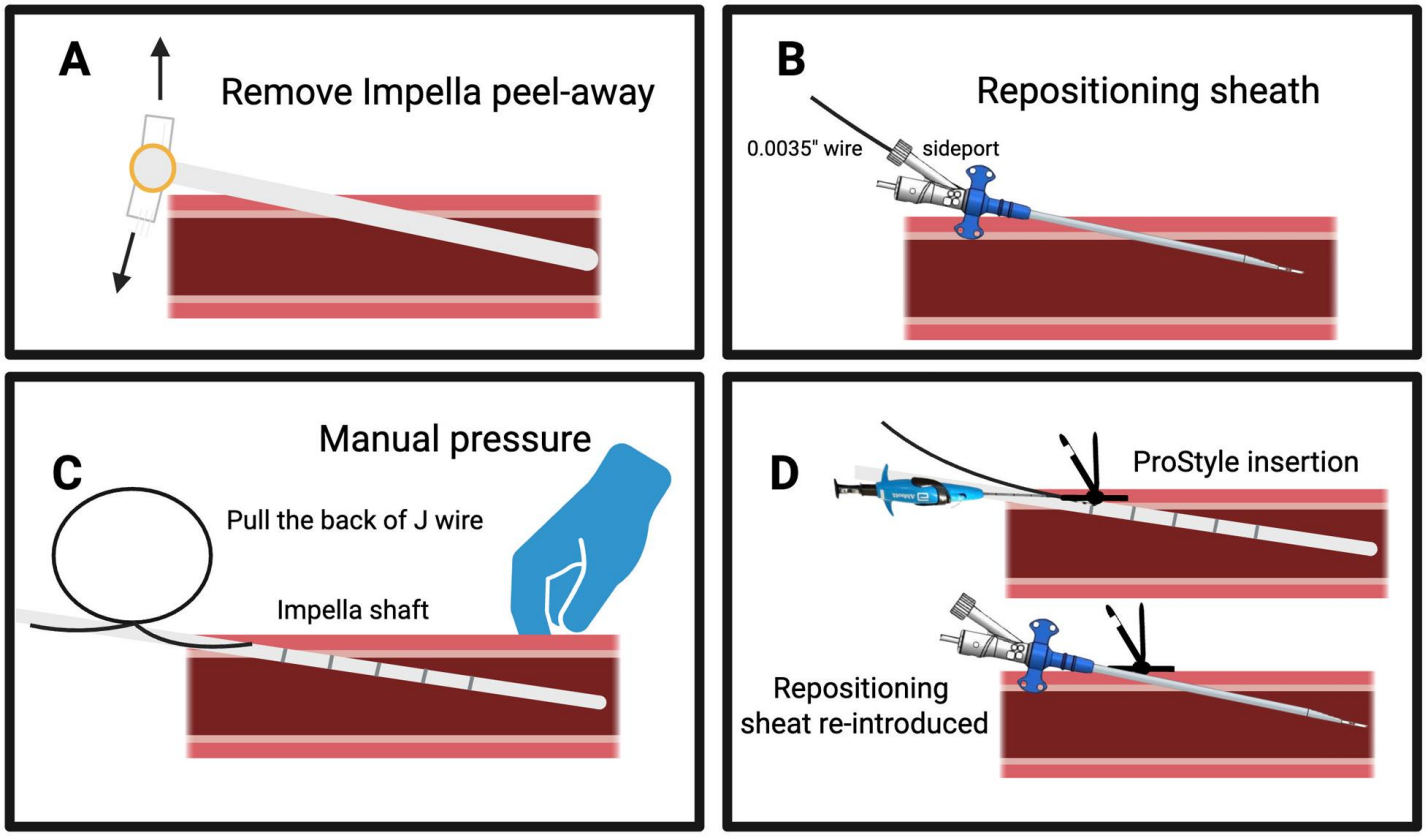
“Sideclose” technique to obtain access closure while maintaining Impella mAFP catheter in place. Panel **(A)**, 14F peel-away sheath removal; Panel **(B)**, Insertion of the repositioning sheath and advancement of a 0.035″ J-wire through the side port; while keeping manual pressure at the arteriotomy site, the repositioning sheath is then withdrawn over the Impella shaft, maintaining the Impella catheter position; Panel **(C)** the backend of the J wire is pulled through the repositioning sheath; Panel **(D)**, the Perclose is advanced over the J-wire, the suture is delivered and the repositioning sheath is reintroduced, and secured by advancing the knot on it.

In patients requiring maintenance of mAFP support after emergent insertion, persistent bleeding around the repositioning sheath may represent a challenge. In addition to the tips to avoid oozing around the cannula described above (e.g., manual pressure, gauze under the sheath to maintain the angle of insertion, gauze soaked in tranexamic acid), a post-closure technique (i.e., “Sideclose” technique) can facilitate hemostasis while maintaining the Impella mAFP catheter in place (44). Briefly, a 0.035″ J-wire is introduced via the side port of the “repositioning sheath”, which is then withdrawn over the mAFP shaft while applying manual compression. The back end of the J-wire is exteriorized, and a Perclose is advanced alongside the mAFP shaft; once deployed, the repositioning sheath is reintroduced and the Perclose suture is tightened, maintaining mAFP function while achieving hemostasis (Figure 7).

### Other post-closure devices and techniques

The collagen-based MANTA 14 Fr device (Teleflex, USA) represents another option for large-bore closure (45, 46). However, randomized comparative evidence—largely derived from transcatheter aortic valve studies—supports the superiority of suture-based devices (47–49), which should therefore be considered the preferred default approach. MANTA device requires prior measurement of arteriotomy depth, which may not be feasible in emergent cases (50). In this context, depth can be assessed using dedicated gauges advanced over the guidewire after femoral puncture, typically applying a small safety margin to the measured value, without the need to open a closure device. In addition, pre-deployment ultrasound assessment is advisable to exclude significant hematoma, which may increase the skin-to-arteriotomy distance and affect depth estimation and closure performance.

**Figure 8:**
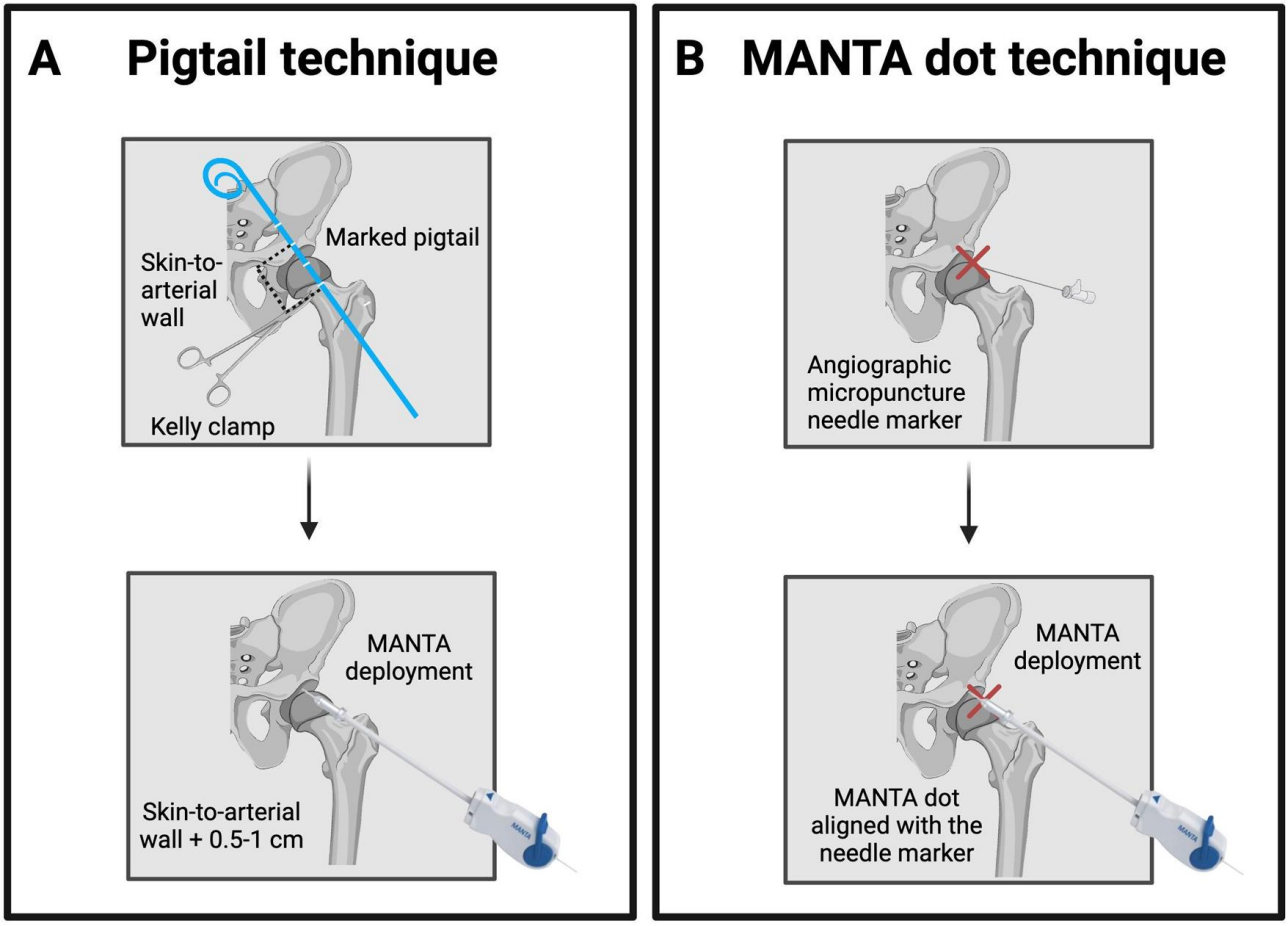
Fluoroscopy post-closure techniques using MANTA (Teleflex, USA) device. Panel **(A)** Pigtail technique. A marked pigtail catheter and Kelly clamp are used to measure the skin-to-arterial wall distance under fluoroscopy. The MANTA device is then deployed at the measured depth plus 0.5–1 cm. Panel **(B)** MANTA dot technique. The arteriotomy site is fluoroscopically marked with a micropuncture needle (red X), and the MANTA radiopaque dot is aligned to this marker during device deployment.

Fluoroscopy-guided adaptations have also been proposed (Figure 8). The first is the marker pigtail catheter technique, using 1 cm markers and a skin clamp under lateral fluoroscopy to estimate depth, with the MANTA deployed at the measured distance plus 0.5–1 cm (51). The second is the “MANTA fluoroscopic dot technique”, which allows avoid depth measurement by fluoroscopically marking the puncture site with a micropuncture needle and aligning the device's radiopaque dot with the arteriotomy site under fluoroscopic guidance (52).

Use of an undersized 8F AngioSeal has also been reported as an alternative post-closure option, often in combination with crossover balloon inflation or manual compression. However, supporting evidence is limited to small series (53). More frequently, AngioSeal is employed as an adjunct to Perclose when hemostasis remains incomplete (26, 39).

PerQseal (Vivasure Medical) is a fully absorbable, sutureless large-bore closure system that deploys an intravascular patch, leaving no permanent implant. Early feasibility studies are evaluating its use for 14Fr mAFP access post-closure (NCT04818541, NCT05951634).

Finally, pledget-assisted hemostasis has been described for residual bleeding after suture-based closure. This involves advancing a surgical polytetrafluoroethylene pledget over the Perclose sutures and securing it with knot-pushers and a slipknot, thereby sealing the residual femoral leak (24, 54).

## Conclusions

Optimizing femoral access and closure techniques is central to safe and effective mAFP use. Vascular complications significantly increase morbidity, mortality, and costs, and their prevention relies on meticulous imaging-guided access, proper device selection, and mastery of both pre-closure and bailout post-closure techniques. A structured, evidence-based approach to access management is therefore essential to improve clinical outcomes of patients undergoing mAFP-supported interventions.

## Supplementary material

Supplementary Video S1 Cross-over technique using an IM guiding catheter, followed by advancement of a 0.018-inch guidewire into the femoral artery alongside the Impella catheter.

Supplementary Video S2 A small contrast injection is performed to confirm vessel patency, followed by advancement of the 0.018-inch guidewire into the superficial femoral artery.

Supplementary Video S3 A 0.035″ guidewire is introduced via the side port of the Impella “repositioning sheath”, maintaining access while catheter is removed in an over-the-wire, rapid-exchange fashion.

Supplementary Video S4 While “dry haemostasis” is maintained with a balloon inflated in the external iliac artery, the 0.035-inch guidewire can be advanced by partially deflating the balloon to allow its passage.

Supplementary Video S5 The Impella device is withdrawn over the wire while rapidly inflating the balloon in the external iliac artery to facilitate hemostasis.

Supplementary Video S6 Angiographic monitoring of Impella catheter removal.

Supplementary Video S7 Balloon inflation facilitates haemostasis during all the steps of post-closure technique using two Perclose.

Supplementary Video S8 Final angiographic control through the tip of the balloon.
